# Effect of Deashing Treatment on Ash Fusion Characteristics of Biochar from Bamboo Shoot Shells

**DOI:** 10.3390/molecules29061400

**Published:** 2024-03-21

**Authors:** Hao Ren, Qi Gao, Liangmeng Ni, Mengfu Su, Shaowen Rong, Shushu Liu, Yanhang Zhong, Zhijia Liu

**Affiliations:** 1 International Centre for Bamboo and Rattan, Beijing 100102, China; hao991105@163.com (H.R.); gaoqi677@163.com (Q.G.); 17346610822@163.com (L.N.); smf13850202763@163.com (M.S.); bramble_001@163.com (S.R.); zyh1842675310@gmail.com (Y.Z.); 2 Key Laboratory of National Forestry and Grassland Administration/Beijing for Bamboo & Rattan Science and Technology, Beijing 100102, China

**Keywords:** bamboo shoot shells, biochar deashing, ash phase transformation, ash fusion

## Abstract

To investigate the influence of deashing on fusion characteristics, a combined method of water and acid washing with different sequences (water washing followed by acid washing, and acid washing followed by water washing) was used to treat the biochar of bamboo shoot shells (BBSSs). The results show that deashing decreased the K content of the biochar from 50.3% to 1.08% but increased the Si content from 33.48% to 89.15%. The formation of silicates and aluminosilicates from alkali metal oxides with silicon was an inevitable result of ash phase transformation at the high temperatures used to improve the fusion temperature (>1450 °C). The thermochemical behavior of ash mainly occurs at 1000 °C. The deashing treatment significantly reduced the reaction intensity during the high-temperature process. This significantly increased the thermal stability of the ash. The adjustment of the washing sequence had a slight impact on the chemical compositions, but the differences in ash micromorphology were obvious. Deashing treatments with different washing sequences can significantly improve ash fusion properties effectively and reduce the risk of scaling, slagging, and corrosion. This study provides a new and reasonable strategy for the deashing of biochar to commercially utilize bamboo shoot shell resources.

## 1. Introduction

As a solid product of biomass pyrolysis, biochar is a renewable, cheap, and sustainable source of energy that has great potential to replace fossil fuels [1]. Combustion is a direct and effective way of using biochar due to its high heating value [2]. Biochar has lower nitrogen and sulfur contents than petroleum fuels, indicating that it releases fewer NO, NO_2,_ and SO_2_ pollutants into the atmosphere [3]. Currently, about 95% of bioenergy is generated by combustion around the world [4]. However, the high contents of alkali metals and alkaline earth metals (AAEMs) often limit the large-scale utilization of biochar as a commercial solid fuel, especially for the alkali metals K and Na. The influence of Ca and Mg on ash fusion characteristics depends on the form of their combination with nonmetallic elements. For example, the existence of CaSO_4_ will increase the fusion temperature of ash, but nonmetallic elements such as S and P are less abundant in biomass materials. Compared with P and S, the influence of Cl is extremely significant. Cl promotes the evaporation of alkalis, which, in turn, leads to the corrosion of boiler heating surfaces. The high content of AAEMs causes boiler scaling and slagging, which reduces the heat transfer efficiency of equipment [5]. Therefore, deashing treatment is necessary to solve the above problems [6], which decreases the ash content of biomass, and changes the proportion of acid–alkali metal oxides to improve the combustion characteristics of biochar.

Washing is a simple and effective method for deashing; water washing and acid washing are the main washing techniques [7]. It is well known that most alkali metals can be removed by water washing due to their good water solubility, and about 10% of acid-soluble alkali metals need to be removed by dilute acid. For example, the ash removal rate of pine bark is less than 10% after water washing, and acid washing can only remove about 30% of K and Na in the bark [8], while the removal rate of Mg and Ca from Ulva lactuca by acid washing is more than 90% [9]. However, some water-soluble AAEMs are transformed into an organic binding form during the pyrolysis process, decreasing the proportion of water-soluble AAEMs in biochar, which significantly reduces the deashing efficiency of water washing [10]. It was confirmed that the removal rate of potassium in biochar by water washing was lower than that in biomass [11,12]. The water-soluble K in biomass is transformed into the stable form of char-K during the pyrolysis process and is further transformed into K-silicates or K-silicaluminates at higher pyrolysis temperatures (above 800 °C). Acid washing solves the above problems because these metals are dissolved by dilute acid. At present, the acids used in pickling ash removal are mainly divided into organic acids, such as acetic acid [13] and citric acid [14], and inorganic acids, including hydrochloric acid [15] and hydrofluoric acid [16]. Compared with organic acid, inorganic acid has stronger acidity and better removal ability for alkali earth metal elements. However, hydrofluoric acid can destroy the C–O–Si structure in biochar to remove most of the Si [17]. Hydrochloric acid is less harmful to the environment than other inorganic acids that are widely used as acid ash removers [18]. It has higher removal rates for alkaline earth metals than water washing, especially for Ca and Mg [19]. Even though acid washing improves deashing efficiency, it increases economic costs and environmental pressure. Therefore, both water washing and acid washing have some limitations in commercial production [20,21]. A feasible strategy is to use a combination of water washing and acid washing for deashing. Such a strategy not only effectively decreases the leaching capacity of AAEMs after biomass pyrolysis, but also reduces the amount of acid to protect the environment and save production costs. All research on deashing has been limited to a single removal method, focusing on the effect of water washing or acid washing. The deashing of biochar by the combination of water and acid washing has not been reported. There is still a lack of sufficient information on the removal efficiency, the fusion characteristics of the ash, and the transformation mechanism of AAEMs. Therefore, it is necessary to systematically investigate the deashing method by combining water and acid washing. 

Bamboo shoot shells (BSSs) are waste in the production process of bamboo shoots. Different from some agricultural wastes, such as rice husks and straw, BSSs have a high moisture content and are rich in peptides and amino acids [22], which makes them perishable and liable to cause environmental pollution [23]. It has been found that biochar from bamboo shoot shells (BBSSs) with a high calorific value has a great application prospect in the field of biomass energy. However, an ash content of 12.67% limits the commercial utilization of BBSSs. In this study, the combination of water and acid washing was adopted to achieve the deashing of BBSSs. Original ash samples were heated to different temperatures, from 700 to 1000 °C, to simulate the repeated heating of the boiler. The purpose of this study was to explore the effects of washing sequences (water washing followed by acid washing and acid washing followed by water washing) on the fusion characteristics and mineral transformation behavior of ash of BBSSs. This study will provide a new and reasonable strategy for the deashing of biochar for the commercial utilization of BSS resources as solid fuels.

## 2. Results and Discussion

### 2.1. Chemical Composition and Fusion Temperature

**Figure 1:**
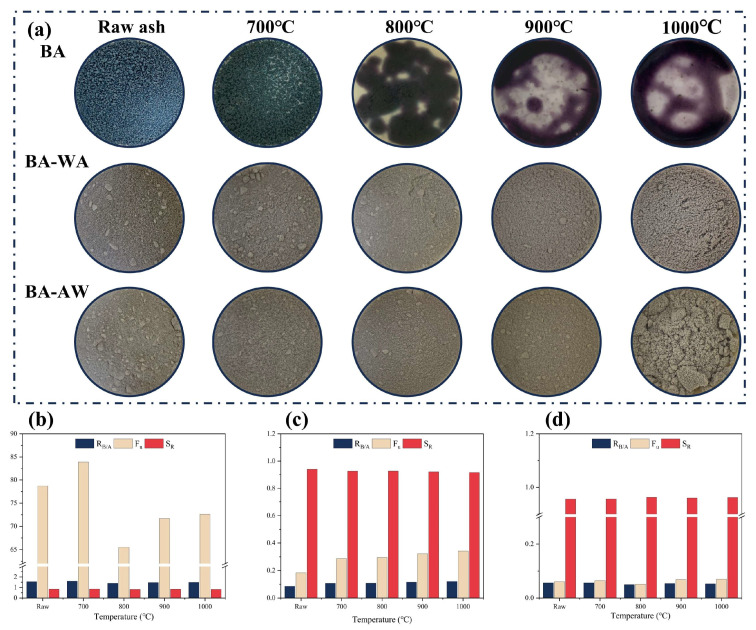
Macroscopic morphology and ash at different temperatures. (**a**): Macroscopic morphology of BA, BA-WA and BA-AW at different temperatures; (**b**): the fusion index of BA; (**c**): the fusion index of BA-WA; (**d**): the fusion index of BA-AW.

Figure 1a shows the appearance characteristics of ash samples at different temperatures. The raw BA presented a dark green color and gradually deepened with the increase in temperatures. When the temperature was up to 800 °C, the ash color changed to purple, indicating the variation in chemical compositions. The BA-700 showed a slight fusion aggregation and adhesion. With the increase in temperature, the fusion degree of BA samples increased gradually. The ash samples obviously melted above a temperature of 800 °C and were attached to the furnace wall after cooling. After deashing, both BA-WA and BA-AW samples showed stable properties during the high-temperature process. They all showed a gray color, and fusion and adhesion phenomena were not found. This confirmed that the fusion characteristics of the BA were significantly improved after deashing. To further explore the fusion behavior, fusion temperatures of BA, BA-WA, and BA-AW were determined, as shown in Table 1. Figure S1 shows the corresponding test images. The BA-WA and BA-AW samples began to deform at a temperature of 1466 °C and 1472 °C, which is obviously higher than the fusion temperature of BA (about 700 °C). This further indicates that the deashing treatment effectively improves the fusion characteristics of BBSS ash. The fusion temperatures of the BA-AW sample were slightly higher than that of the BA-WA sample. The fusion behavior of ash was closely related to temperature, but its composition played an important role [24]. Therefore, the chemical compositions of ash at different temperatures were further investigated in this research.

The chemical components of ash at different temperatures are shown in Table 2. The scaling and slagging indexes are shown in Figure 1b–d, which were calculated according to the ash composition of ash samples. Table 1 shows that deashing significantly decreased the ash content of BBSS. The ash content of BBSS-WA was 1.41% and that of BBSS-AW was 2.78%. The main components of BA were K and Si. Potassium oxide and silicon dioxide were representative basic and acidic oxides in biomass ash, which were the key factors affecting the fusion characteristics of ash [25]. It is well known that the existence of acid oxides increases the ash fusion temperature and decreases the risk of boiler fouling and slagging. On the contrary, the presence of alkali metals, such as K and Na, reduces the fusion temperature and increases the risk of corrosion and scaling in the boiler. After deashing, Si and K elements occurred with a variation. Most K elements in BBSS ash were water-soluble and acid-soluble and were efficiently removed by the combination of water washing and acid washing. The remaining insoluble K was in the form of potassium silicate, which did not exist in the biomass itself [26]. In addition, the removal rate of the main elements was calculated under different washing sequences, as shown in Table 3. Both BA-WA and BA-AW had higher removal rates of ash elements. It is worth noting that there was an obvious difference in the removal rates of Si. Si elements were soluble in an alkaline environment. When the BBSSs were first washed with water, the washing solution was alkaline, resulting in partial dissolution of Si. Therefore, the Si content in BA-AW was higher than that in BA-WA. Furthermore, Cl was used as an auxiliary agent for alkali metal volatilization, and alkali metal chloride condensed on the heat transfer surface of the boiler, resulting in corrosion [4]. Deashing decreased the Cl content of BBSS from BA of 1.63% to BA-WA of 0.07% and BA-AW of 0.03%. At high temperatures, Cl and K in gaseous form were released. The release path of AAEMs was not only limited to the sublimation of chloride, but also included the dissociation of other salts, such as carbonate, phosphate, sulfate, and silicate, especially for biomass-based materials with low Cl content [27]. The P content in BA and the S content in BA-WA and BA-AW gradually decreased with the increase in temperatures, which was attributed to the thermal decomposition of corresponding alkali salts [28]. Comparing the chemical components in the ash with different washing sequences, Table 2 shows that the influence of washing sequences on chemical components was slight. After deashing, most of the ash components were silicon, which significantly improved the fusion characteristics of ash. According to the standard deviation analysis, the relative standard deviation (RSD) of SiO_2_ in the BA was 7.88%, while that of BA-WA and BA-AW was 0.34% and 0.20%. This was because BA contains a lot of AAEM oxide, which has strong water absorption [29]. However, the influence of silicon on ash fusion temperature needs to be further explored. The reactions and transformation of ash at high temperatures are complicated, and the variation in each chemical component cannot explain the chemical reaction.

To further clarify the impact of deashing treatment on scaling and slagging risk, the acid–base ratio index (R_b/a_), fouling index (F_u_), and slag viscosity index (S_R_) were calculated according to each chemical component of ash, as shown in Figure 1b–d. Figure 1b confirms that the value of R_b/a_ was higher than 1 [30], and the F_u_ value was higher than 40 [31], indicating a high slagging tendency of ash. The S_R_ index was higher than 0.78 [32], confirming a low slagging tendency. However, the influence of alkali metals was not considered in the calculation of S_R_ [33]. BA-WA (Figure 1c) and BA-AW (Figure 1d) had a low slagging tendency, which was consistent with the thermal behavior of ash at high temperatures (Figure 1a). Compared with previous studies, the combination of acid and water washing helped to improve ash fusion characteristics. Overall, the ash content of BBSSs was significantly reduced under different treatment methods. The variation in chemical compositions increased the fusion temperature of ash to avoid slagging and corrosion on thermal conversion equipment. This was beneficial to convert BBSSs to fuels. In addition, the ash content was also an important index in the field of commercial biological activated carbon production. After deashing treatment, the ash content of BBSSs was decreased to 1.41%. This result provided the possibility for BBSSs to be used as a raw material for the preparation of commercial activated carbon.

### 2.2. Transformation Behavior of Minerals

**Figure 2:**
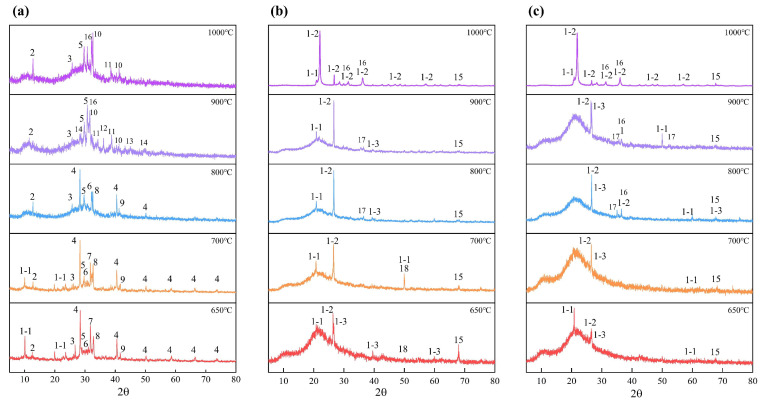
Mineral transformation behavior of BA (**a**), BA-WA (**b**), and BA-AW (**c**). Annotation: 1-1—tridymite, 1-2—cristobalite, 1-3—moganite, 2—AlPO_4_, 3—CaO, 4—KCl, 5—K_2_SO_4_, 6—K_2_CO_3_, 7—CaCO_3_, 8—MgCO_3_, 9—CaSO_4_, 10—CaSiO_3_, 11—K_2_SiO_3_, 12—CaCl_2_, 13—K_2_CaSiO_4_, 14—K_2_O, 15—Al_2_O_3_, 16—MgSiO_3_, 17—Mg_2_SiO_4_, 18—MgO.

The fusion behavior and characteristics of ash samples were affected by the mineral composition and content. To explore the mineral evolution of ash samples at high temperatures, XRD was used to characterize the mineral composition of ash samples. The mineral transformations of BA, BA-WA, and BA-AW prepared at different temperatures are shown in Figure 2a–c. Figure 2a shows that the minerals of BA were mainly composed of KCl, SiO_2_, CaCO_3_, K_2_SO_4_, K_2_CO_3_, and MgCO_3_. Among them, the obvious diffraction peak of KCl indicates that the chlorine element in BA mainly exists in the form of KCl. With the increase in temperatures, the diffraction peak of KCl decreased gradually, because the high temperature promoted the gasification of KCl (Equation (1)). The presence of CaCl_2_ at a temperature of 900 °C confirms that a small amount of Cl also exists in the form of CaCl_2_. The potassium not only exists in the form of chloride but the corresponding carbonate and sulfate were also found. The K_2_CO_3_ diffraction peak disappeared above a temperature of 900 °C because it decomposed to form K_2_O (Equation (2)). Accordingly, K_2_O was found in the BA-900 diffraction peak. The diffraction peak intensity of K_2_SO_4_ increased gradually with the increase in temperatures because of its high pyrolysis temperature of 1000 °C. CaO was found in the diffraction peak of 650 °C. CaCO_3_ completely decomposed into CaO at a temperature of 800 °C (Equation (3)). When the temperature was up to 700 °C, MgCO_3_ began to decompose and release CO_2_ (Equation (4)). Therefore, the diffraction peak of MgCO_3_ disappeared at a temperature of 900 °C. The Si was the most abundant element, except for K in BA, which mainly existed in the form of SiO_2_ at low temperatures. It maintained stability at a lower temperature (˂800 °C). When the temperature was 900 °C, the stability of the longer chemical bond formed between K and oxygen atoms was weakened to integrate K into the silicon–oxygen system. Therefore, SiO_2_ reacted with the K_2_O to form the corresponding K_2_SiO_3_. The formation of other silicates was similar, such as CaSiO_3_, and MgSiO_3_ (Equations (5)–(7)). At higher temperatures, Al–O with higher stability was added to the reaction of this system [34]. Thus, the intensity of the SiO_2_ diffraction peak reduced and gradually disappeared, accompanied by the formation of a silicate diffraction peak. The diffraction peaks of AlPO_4_ and CaSO_4_ were also found. AlPO_4_ had strong thermal stability, which did not cause thermal decomposition or crystal phase transformation when the temperature was lower than 1000 °C. The pyrolysis temperature of CaSO_4_ was about 1200 °C [35], which was stable below a temperature of 1000 °C. The increase in diffraction peaks at a temperature of 900 °C and 1000 °C was attributed to the superposition of CaSO_4_ and CaSiO_3_.

(1) $$\mathrm{KCl}\;\left(s\right)\to \mathrm{KCl}\;\left(g\right)$$

(2) $$K_{2}{\mathrm{CO}}_{3}\to K_{2}O+{\mathrm{CO}}_{2}$$

(3) $${\mathrm{CaCO}}_{3}\to \mathrm{CaO}+{\mathrm{CO}}_{2}$$

(4) $${\mathrm{MgCO}}_{3}\to \mathrm{MgO}+{\mathrm{CO}}_{2}$$

(5) $$K_{2}O+{\mathrm{SiO}}_{2}\to K_{2}{\mathrm{SiO}}_{3}$$

(6) $$\mathrm{CaO}+{\mathrm{SiO}}_{2}\to {\mathrm{CaSiO}}_{3}$$

(7) $$\mathrm{MgO}+{\mathrm{SiO}}_{2}\to {\mathrm{MgSiO}}_{3}$$

Compared with BA, the mineral compositions of BA-WA and BA-AW only appeared in SiO_2_, Al_2_O_3,_ and MgO diffraction peaks at lower temperatures, because acid and water washing removed the AAEMs from ash samples. The crystal form of SiO_2_ was divided into tridymite, cristobalite, and moganite [36]. The crystal form of SiO_2_ varied with the increase in temperatures. Tridymite and moganite were transformed into cristobalite structures, and this behavior was likely to occur in the presence of alkali metals. In the silica molecule, the silicon–oxygen bond was a kind of polar bond with the characteristics of the covalent bond, and the covalent bond structure was still stable at high temperatures. The crystal structure of silicon dioxide was a three-dimensional network structure formed by tetrahedral silicon ions and oxygen ions with high strength and stability, which made silicon dioxide own a high fusion point. This was also the reason why the increase in silicon content improved the fusion temperature of ash. Compared with BA-WA and BA-AW, there was an obvious diffraction peak of moganite in BA-AW at a temperature of 900 °C. The removal of alkali metal elements in BA-AW increased the conversion temperature of moganite. In the process of high-temperature fusion, MgO and SiO_2_ formed Mg_2_SiO_4_ (2MgO·SiO_2_). When the temperature further increased, the silicon magnesium compound was mainly in the form of MgSiO_3_ because the molecular structure of silica also existed in silicate molecules with high fusion temperatures. It is well known that the existence of silicate improves the fusion temperature of ash, except for calcium silicate, which is regarded as a cosolvent mineral [4]. Because of the remarkable effect of acid washing on calcium removal, the existence of calcium silicate was only found in BA. Alumina (corundum) did not participate in chemical reactions below temperatures of 1000 °C because of its strong thermal stability. However, the formation of aluminosilicate was found at the high temperatures [37]. Furthermore, the X-ray diffraction pattern (2θ = 15–30°) of BA-WA and BA-AW had a wide diffraction peak in the low-temperature range, because the ash was amorphous at a temperature lower than 900 °C. Silica crystals with higher crystallization strengths were precipitated when the temperature was higher than 900 °C [38].

It is well known that a number of AAEMs (mainly potassium) exist in the form of low fusion point chloride, carbonate, and sulfate, which decrease the fusion temperature of ash. The high temperature made chlorides (KCl) and carbonates evaporate and thermally decompose. Sulfates also began to decompose to form corresponding AAEM oxides, carbon dioxide, and sulfur dioxide. Furthermore, the AAEM oxides combined with SiO_2_ to form silicate. Compared with a single reaction system, these reactions were easier to take in a system with complex chemical compositions, where a large number of alkali metals existed. Inorganic types in biomass materials were divided into three types: water-soluble, acid-soluble, and other residual parts. Therefore, the water and acid washing removed these key inorganic metal ions to reduce the formation of corresponding salts and the scaling and slag risk of boilers. 

### 2.3. Synchronous Thermal Analysis

**Figure 3:**
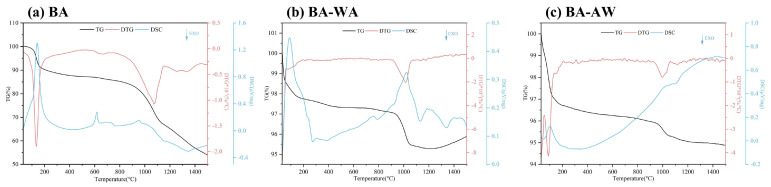
TG, DTG, and DSC curves of ash samples.

The fusion characteristics and chemical changes of ash at high temperatures can be accurately analyzed by recording the mass and heat flow variation. Figure 3 shows the TG, DTG, and DSC curves of ash samples. At a temperature of 100 °C, the TG–DTG curves of BA, BA-WA, and BA-AW showed obvious weight loss peaks, and the corresponding heat absorption peaks were also found in the DSC curves. Because ash had a certain hygroscopicity, the changes at this stage were mainly caused by the evaporation of water. However, the temperature range of BA was higher than that of BA-WA and BA-AW due to the higher water evaporation resistance from the complexity of hydrated compounds in BA. The KHCO_3_ in BA underwent thermal decomposition to form K_2_CO_3_ at a low temperature to release CO_2_ and H_2_O. The first stage of BA ended at a temperature of around 200 °C. There was a weight loss peak in the DTG curve and a sharp endothermic peak in the DSC curve at a temperature of 615 °C, indicating there was an accelerated endothermic process because of the thermal decomposition of CaCO_3_ (Equation (3)). A wide and fuzzy heat absorption peak was found in the DSC curve at a temperature of about 700 °C, where BA began to transform from a solid state to a molten state. With the increase in temperatures, the fusion behavior became increasingly intense, which was consistent with the macro change of ash in Figure 1. Furthermore, MgCO_3_ also occurred to thermal decomposition at this stage (Equation (4)) [39]. KCl began to transform into the gas phase when the temperature reached its fusion point of 776 °C [40]. This is the main release mode of Cl. When the temperature was lower than the fusion point, Cl was mainly released in the form of HCl [27]. This phenomenon confirmed that the Cl content of BA-700 was higher than that of BA. According to the XRD results, this behavior continued up to a temperature of 900 °C (Equation (1)) [41]. Based on the results of XRF, its main volatilization temperature was from 800 °C to 900 °C. At a temperature of 800 °C, the weight loss rate of ash samples increased gradually. At this stage, the volatilization of KCl, and the fusion and thermal decomposition of K_2_CO_3_ were found (Equation (2)). The maximum weight loss peak was found at a temperature of 900–1100 °C. There was an endothermic peak at a temperature of 950 °C due to the fusion and thermal decomposition of K_2_SO_4_ (Equation (8)) according to the changes in the chemical compositions of BA. The fusion point of K_2_SO_4_ was at a temperature of 1071 °C. The fusion behavior of K_2_SO_4_ was changed due to the existence of low fusion point compounds, such as KCl and K_2_CO_3_ [42]. For the same reason, the formation of silicates occurred at a temperature of 900 °C. The evaporation and decomposition of salt occurred at a wider temperature range. At a temperature of 1200 °C, the thermal decomposition of K_2_CO_3_ and K_2_SO_4_ resulted in the persistent weight loss of ash samples. The SiO_2_ lost its stability and reacted with the K to form potassium silicoaluminate at higher temperatures [43]. Because the content of K in BA was much higher than that of Cl, K in BA was not only in the form of KCl. According to the TG–DSC curves, it was concluded that the main reaction during the main weightlessness stage was the thermal decomposition of K_2_CO_3_. 

Deashing removed a large amount of Ca from the ash samples. The XRF data showed that the Ca content of BA-WA was twice as much as that of BA-AW. Therefore, the DSC curve of BA-WA had an obvious endothermic peak of CaCO_3_ pyrolysis, which disappeared at temperatures of 600–800 °C. Compared with BA, deashing removed the chemical compositions of BA-WA and BA-AW. Silicon accounted for about 82–90% of the compositions of ash samples. Acid washing could not remove sulfates, such as K_2_SO_4_, MgSO_4_, and CaSO_4_. The maximum weight loss peak of BA-WA and BA-AW was at a temperature of around 1000 °C because of the thermal decomposition of sulfate, accompanied by the overflow of SO_2_ and O_2_ (Equations (8)–(10)). The weight loss rate at this stage was less than 2%, confirming that most of the AAEMs in BBSSs existed in the form of carbonate and were easily removed by a combination of water and acid washing. The appearance of endothermic peaks between a temperature of 1100 °C and 1400 °C was due to the formation of silica aluminate (Equations (11)–(13)) [44]. At a temperature of 1450 °C, both BA-WA and BA-AW had an endothermic peak due to the fusion phenomenon when the pyrolysis temperature was up to the deformation temperature of ash [45]. The mass loss of BA was about 36% (excluding water volatilization), while that of BA-WA and BA-AW were 2.47% and 1.9%. This also confirmed that the deashing treatment significantly improved the thermal stability of ash.

(8) $$K_{2}{\mathrm{SO}}_{4}\to K_{2}O+{\mathrm{SO}}_{2}+\frac{1}{2}O_{2}$$

(9) $${\mathrm{MgSO}}_{4}\to \mathrm{MgO}+{\mathrm{SO}}_{2}+\frac{1}{2}O_{2}$$

(10) $${\mathrm{CaSO}}_{4}\to \mathrm{CaO}+{\mathrm{SO}}_{2}+\frac{1}{2}O_{2}$$

(11) $$2\mathrm{MgO}+{\mathrm{Al}}_{2}O_{3}+{\mathrm{SiO}}_{2}\to {\mathrm{Mg}}_{2}{\mathrm{Al}}_{2}{\mathrm{SiO}}_{7}$$

(12) $$2\mathrm{CaO}+{\mathrm{Al}}_{2}O_{3}+{\mathrm{SiO}}_{2}\to {\mathrm{Ca}}_{2}{\mathrm{Al}}_{2}{\mathrm{SiO}}_{7}$$

(13) $$K_{2}O+{\mathrm{Al}}_{2}O_{3}+2{\mathrm{SiO}}_{2}\to 2{\mathrm{KAlSiO}}_{4}$$

### 2.4. Micro-Morphology of Minerals

**Figure 4:**
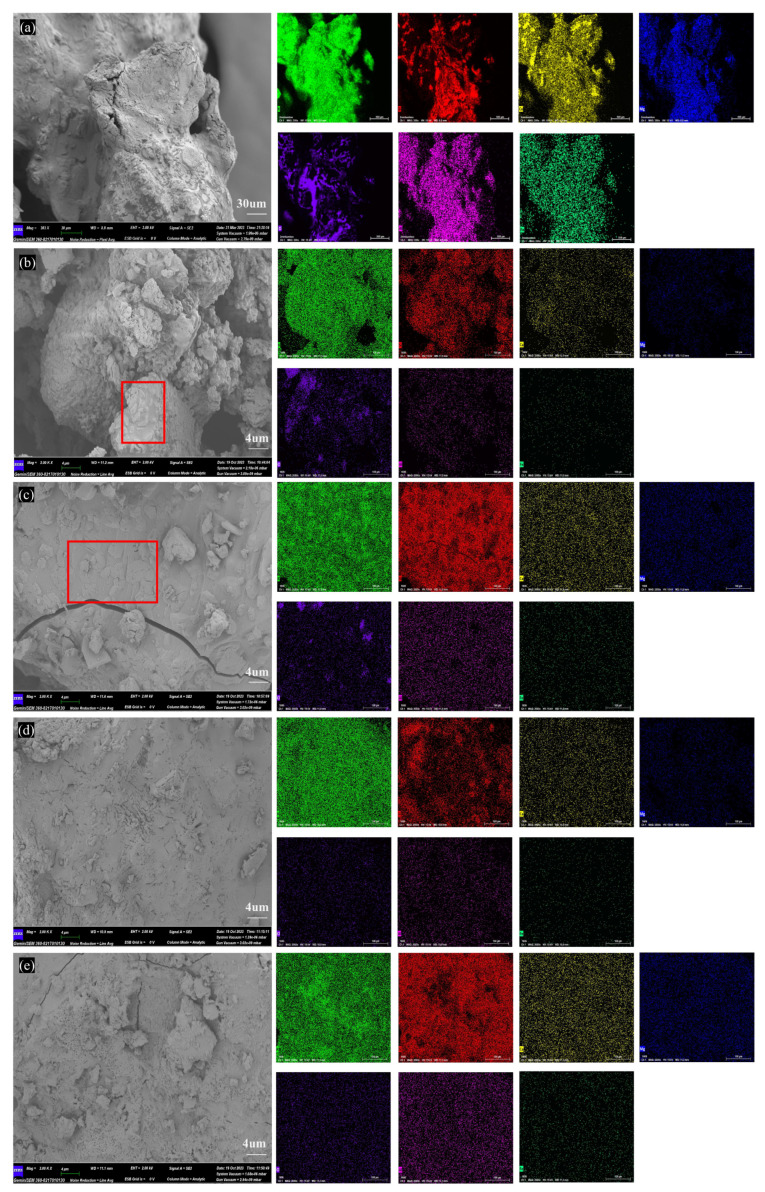
SEM-EDS images of BA at different temperatures. (**a**–**e**) Micro-topography of BA, BA-700, BA-800, BA-900, BA-1000. Annotation: red square in subfigure (**b**) is unmelted ash blocks; red square in subfigure (**c**) is the unmelted ash blocks occurred to fusion behavior.

Figure 4 shows the microstructure changes in BA at different temperatures. The microstructure of BA-WA and BA-AW are shown in Figures S2 and S3. Figure 4b confirms that the ash sample of BA-700 had an incomplete fusion phenomenon, and the unmelted ash blocks were found. Figure 4c indicates that the unmelted bulk part of BA-700 occurred to fusion behavior when the temperature reached 800 °C, and completely melted at temperatures of 900 °C and 1000 °C (Figure 4d,e). This was consistent with the change in macro morphology with the increase in temperatures. Figure 4e confirms that there was a dispersed block structure on the surface of the ash sample due to the overflow of a large number of volatile substances or decomposition products at high temperatures. Table 4 shows the element contents of the BA, BA-WA, and BA-AW at different temperatures. In the molten system of BA, the K content of ash samples was up to 66%, followed by Si and Ca. The Cl content was significantly reduced at high temperatures, confirming that KCl was volatilized after a temperature of 700 °C. The KCl was completely released at a temperature of 900 °C. Deashing significantly varied the chemical compositions of ash, resulting in Si content being the highest. The ash skeleton is mainly composed of silicon to improve the thermal stability of BA-WA and BA-AW.

**Figure 5:**
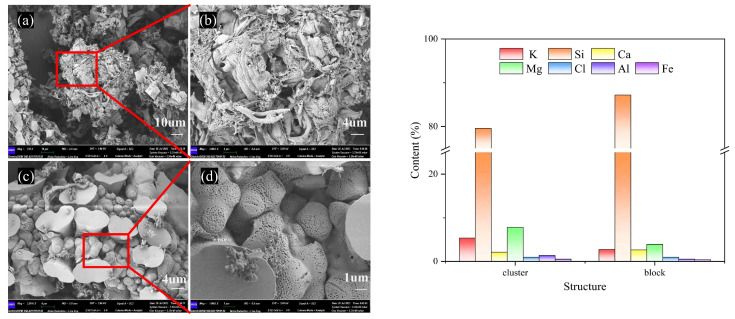
SEM images and EDS results of BA-WA. (**a**,**c**) are the characteristic diagrams of row-like clustered and irregular block structures in BA-WA, respectively. (**b**,**d**) are enlarged images of (**a**) and (**c**), respectively.

According to the micro-morphology of BA-WA and BA-AW (Figures S1 and S2), there were only two characteristics of the row-like cluster structure and the irregular block structure in BA-WA and BA-AW. Figure 5a–d show that there were dense pores on the surface of the ash skeleton in the microstructure of BA-WA, which were not found in BA-AW. This is due to the thermal decomposition of the alkali salts which were insoluble in water and acid of BA-WA at high temperatures, caused by the overflow of decomposition gas. Table 4 indicates that the surface alkali content of BA-WA was higher than that of BA-AW because there were no dense pores in the ash skeleton in BA-AW. Due to the low fusion point of BA, ash exists in the form of a molten body at high temperatures. There will be no similarly dense pores on the surface of BA because a number of alkali metal salts were decomposed at high temperatures. Figure 5 shows a certain difference in the element content of the two structures. The variation in potassium and magnesium was significant. However, the content of silicon changed slightly.

## 3. Material and Methods

### 3.1. Raw Materials

BSSs of moso bamboo (*Phyllostachys edulis*) were taken from Nanping City, Fujian Province, China. They were washed off any soil with deionized water and pulverized using a crusher (Lingsum-1000C, Lingsum, Hefei, China) after drying. The particles of 250 to 380 µm were collected and dried in the oven at a temperature of 102 ± 3 °C for 12 h. The BBSSs were prepared using a carbonization furnace (SN 333301, Nabertherm, Bremen, Germany) under a nitrogen atmosphere, which was heated from room temperature to 450 °C with a heating rate of 5 °C/min.

Hydrochloric acid (HCl, AR, 37%) was purchased from Aladdin, Shanghai, China. It was diluted to a concentration of 1 mol/L with deionized water. Deionized water was prepared by an ultrapure water machine (EIKEY, AK-RO-C2, Jinan, China).

### 3.2. Deashing of BBSS

The deashing of BBSSs was carried out by a combination of water washing and acid washing with different sequences (water washing followed by acid washing, or acid washing followed by water washing). For water washing followed by acid washing, 10 g BBSS was soaked in the 300 mL water solution and stirred by a stirrer (IKA, EURO–ST40 KLC DS025, Staufen, Germany) at a speed of 200 r/min for 4 h. Then, the BBSS was separated from the washing solution by vacuum filtration (LC–VWP–60T, LICHEN, Shanghai, China) and further washed using deionized water until the pH value of the filtrate was 7.0. It was transferred to the 300 mL HCl solution of 1 mol/L and further stirred for 3 h. Then, it was filtered out using vacuum filtration and washed using deionization water until the pH value of the filtrate was 7.0. The washed BBSS was dried in the oven at a temperature of 102 ± 3 °C for 24 h and labeled BBSS-WA. For acid washing followed by water washing, 10 g BBSS was soaked in the 300 mL HCl solution of 1 mol/L and stirred by a stirrer at a speed of 200 r/min for 3 h. The BBSS was separated from the washing solution by vacuum filtration and washed using deionized water until the pH value of the filtrate was 7.0. It was transferred to the 300 mL water solution and further stirred for 4 h. Then, it was filtered out using vacuum filtration and washed using deionization water until the pH value of the filtrate was 7.0. The final BBSS was labeled BBSS-AW. In this process, temperatures of water washing and acid washing were set to 75 °C.

### 3.3. Preparation of Ash Samples

The ash samples including BBSS ash (BA), BBSS-WA ash (BA-WA), and BBSS-AW ash (BA-AW) were prepared using a muffle furnace in the atmosphere. They were heated from room temperature to 650 °C with a heating rate of 10 °C/min for 5 h. They were respectively heated to the target temperature of 700 °C, 800 °C, 900 °C, and 1000 °C for 2 h in the air atmosphere to ensure a complete reaction. After cooling to room temperature, the ash samples were collected and labeled to BA-700, BA-800, BA-900, and BA-1000 according to target temperatures and the macroscopic morphology was recorded with a camera (Canon EOS R50, Tokyo, Japan)

### 3.4. Characterization of Ash Samples

The microstructure and element distribution of ash samples were investigated by a Scanning Electron Microscope (SEM, Gemini–SEM360, Zeiss, Oberkochen, Germany) with an Energy Dispersive Spectrometer (EDS, X–flash Detector 630M, Bruker, Billerica, MA, USA). X-ray fluorescence spectroscopy (XRF, X–supreme8000, Oxford instrument, Oxford, UK) and X-ray diffraction (XRD, D2 PHASER XE–T, Bruker, Billerica, MA, USA) were used to determine the elements and mineral composition. The absolute content (A_x_) and removal rate (D_x_) of each ash-forming element were calculated according to Equations (14) and (15). The diffraction angle ranged from 5° to 90°, and the scanning speed was 0.02°/s. The crystal phase of compounds was analyzed by JADE 6.5 software.

(14) $$A_{X}=C\times E_{X\cdot O}\times M_{X}$$

(15) $$D_{X}=\left(A_{X\cdot \mathrm{BSSC}}-A_{X\cdot \mathrm{deashing}\;\mathrm{BSSC}}\right)/A_{X\cdot \mathrm{BSSC}}$$

where C is the ash content of BBSS, E_x·O_ is the oxide content of ash-forming elements from XRF, M_x_ is the mass percentage of ash-forming elements in the corresponding oxides.

### 3.5. Determination of Thermal Behaviors

The thermal behaviors of ash samples were performed using a synchronous thermal analysis (TGA/DSC3, Mettler-Toledo, Zurich, Switzerland). About 20–25 mg ash samples were heated from room temperature to 1500 °C under a nitrogen atmosphere with a heating rate of 10 °C/min. The gas flow rate was 50 mL/min. 

### 3.6. Determination of Fusion Properties

According to the GB/T219-2008 [46], fusion temperatures of ash samples were determined by an automatic ash fusion analyzer (TJHR–6000, Tianjian Technology, Hebi, China) in a reducing atmosphere (CO_2_:CO = 4:6, Volume ratio). The ash sample was heated to 900 °C at a heating rate of 15 °C/min, and then further heated to 1500 °C at a heating rate of 5 °C/min. The deformation temperature (DT), softening temperature (ST), hemispheric temperature (HT), and flow temperature (FT) were determined based on the shape of the ash cone.

## 4. Conclusions

Deashing varied the content and chemical compositions of ash. It removed an amount of K, Cl and Ca to make Si account for about 82–90% of the chemical composition in ash. The minerals of BA were composed of KCl, SiO_2_, CaCO_3_, K_2_SO_4_, K_2_CO_3_, and MgCO_3_. However, BA-WA and BA-AW mainly included SiO_2_, Al_2_O_3,_ and MgO. This significantly improves fusion characteristics to increase the fusion temperature of 700 °C for BA to a temperature of 1466 °C for BA-WA and 1472 °C for BA-AW. The adjustment of washing order had a slight influence on fusion properties. The combination of water and acid washing will be an effective and feasible method to improve the scaling and slagging risk of ash and promote commercial utilization of BBSSs as bioenergy products.

## Figures and Tables

**Table 1:** Ash yield and ash fusion temperature of samples.

Samples	Content (%)	DT (°C)	ST (°C)	HT (°C)	FT (°C)
BA	12.67	<900	<900	<900	<900
BA-WA	1.41	1466	1485	1489	1492
BA-AW	2.78	1472	1489	>1500	>1500

**Table 2:** Chemical composition of ash samples.

Samples	Chemical Composition (wt%)									
	MgO	Al_2_O_3_	SiO_2_	P_2_O_5_	SO_3_	Cl	K_2_O	CaO	TiO_2_	Fe_2_O_3_
BA	1.62 ± 0.10	3.07 ± 0.21	33.48 ±2.64	2.05 ± 0.04	1.98 ± 0.03	1.63 ± 0.05	50.30 ± 1.52	2.67 ± 0.21	0.17 ± 0.01	1.76 ± 0.09
BA-700	1.48 ± 0.08	2.78 ± 0.22	32.82 ±1.73	1.83 ± 0.02	1.99 ± 0.05	2.16 ± 0.10	51.65 ± 1.68	2.28 ± 0.07	0.16 ± 0.01	1.68 ± 0.09
BA-800	1.82 ± 0.08	3.24 ± 0.10	35.47 ±1.56	1.61 ± 0.01	2.14 ± 0.02	1.54 ± 0.04	46.17 ± 1.23	3.67 ± 0.09	0.24 ± 0.01	2.56 ± 0.01
BA-900	1.36 ± 0.04	3.21 ± 0.20	34.54 ±2.61	1.93 ± 0.07	2.99 ± 0.04	0.55 ± 0.03	48.10 ± 1.70	3.13 ± 0.15	0.19 ± 0.01	2.25 ± 0.06
BA-1000	1.18 ± 0.03	3.35 ± 0.07	34.44 ± 1.52	1.65 ± 0.03	2.20 ± 0.01	0.31 ± 0.01	48.81 ± 1.36	3.60 ± 0.02	0.26 ± 0.01	3.01 ± 0.11
BA-WA	3.96 ± 0.17	2.74 ± 0.10	83.46 ± 0.28	1.56 ± 0.04	2.10 ± 0.04	0.07 ± 0.002	2.67 ± 0.07	1.53 ± 0.08	0.49 ± 0.03	0.64 ± 0.03
BA-WA-700	4.51 ± 0.12	3.49 ± 0.18	82.08 ± 0.19	1.73 ± 0.03	2.13 ± 0.03	0.05 ± 0.002	2.66 ± 0.06	1.41 ± 0.06	0.50 ± 0.02	0.68 ± 0.03
BA-WA-800	4.30 ± 0.21	3.33 ± 0.14	82.34 ± 0.28	1.77 ± 0.04	1.86 ± 0.05	0.03 ± 0.003	2.74 ± 0.10	1.54 ± 0.04	0.54 ± 0.02	0.73 ± 0.02
BA-WA-900	4.60 ± 0.18	3.17 ± 0.05	82.76 ± 0.62	1.79 ± 0.06	0.78 ± 0.2	0.02 ± 0.0006	2.80 ± 0.07	1.68 ± 0.16	0.56 ± 0.02	0.86 ± 0.08
BA-WA-1000	4.81 ± 0.07	2.85 ± 0.06	82.62 ± 0.22	1.85 ± 0.04	0.70 ± 0.005	0.02 ± 0.002	2.82 ± 0.07	1.99 ± 0.08	0.47 ± 0.02	0.78 ± 0.03
BA-AW	2.93 ± 0.03	2.48 ± 0.12	89.15 ± 0.18	1.14 ± 0.03	1.31 ± 0.04	0.03 ± 0.001	1.08 ± 0.04	0.77 ± 0.03	0.27 ± 0.01	0.37 ± 0.01
BA-AW-700	2.86 ± 0.08	2.55 ± 0.09	88.94 ± 0.21	1.20 ± 0.08	1.29 ± 0.07	0.03 ± 0.002	1.14 ± 0.03	0.77 ± 0.02	0.31 ± 0.01	0.39 ± 0.01
BA-AW-800	2.48 ± 0.05	2.25 ± 0.08	90.34 ± 0.04	1.00 ± 0.04	1.07 ± 0.04	0.03 ± 0.002	1.04 ± 0.01	0.70 ± 0.01	0.25 ± 0.004	0.35 ± 0.005
BA-AW-900	2.52 ± 0.04	2.56 ± 0.15	89.80 ± 0.14	1.11 ± 0.09	0.63 ± 0.04	0.02 ± 0.002	1.26 ± 0.02	0.79 ± 0.02	0.31 ± 0.01	0.43 ± 0.01
BA-AW-1000	2.24 ± 0.11	2.12 ± 0.07	90.23 ± 0.55	1.23 ± 0.11	0.56 ± 0.05	0.02 ± 0.002	1.32 ± 0.04	0.85 ± 0.08	0.31 ± 0.02	0.48 ± 0.07

**Table 3:** Removal rate of main elements in ash.

	Removal Rate of Main Elements (wt%)						
	K	Si	Cl	Ca	Mg	Fe	Al
BA-WA	99.41	72.24	99.52	93.62	72.78	95.95	90.06
BA-AW	78.04	41.53	99.60	93.67	60.28	95.38	82.26

**Table 4:** EDS results of BA in different temperatures.

Samples	K	Si	Ca	Mg	Cl	Al	Fe
BA	72.18	14.95	2.25	0.71	8.58	0.40	0.05
BA-700	59.72	28.90	1.62	0.75	2.32	0.57	1.29
BA-800	55.69	35.36	2.61	1.21	1.91	0.68	1.44
BA-900	66.45	23.52	2.54	0.22	1.21	0.14	0.69
BA-1000	60.79	28.29	3.73	0.90	0.44	0.52	1.72
BA-WA	4.98	87.23	3.17	2.56	0.24	0	1.02
BA-WA-700	1.60	92.39	0.46	1.66	0	1.31	0.03
BA-WA-800	1.87	92.12	0.67	2.58	0.01	0.77	0.28
BA-WA-900	3.92	80.89	1.56	9.22	0	1.53	0.28
BA-WA-1000	1.81	87.55	1.34	5.96	0	0.59	0.14
BA-AW	0.58	96.55	0.98	1.17	0.10	0.35	0.12
BA-AW-700	0.50	95.7	0.07	0.51	0.15	0.22	0.05
BA-AW-800	1.35	88.09	0.44	4.51	0.06	0.55	0.35
BA-AW-900	0.56	93.05	0.44	3.73	0	0.41	0.21
BA-AW-1000	0.99	92.62	0.70	1.83	0.05	1.05	0.33

## Data Availability

Data are contained within the article and Supplementary Materials.

## Supplementary Materials

The following supporting information can be downloaded at: https://www.mdpi.com/article/10.3390/molecules29061400/s1, Figure S1: Ash fusion temperature of BA-WA and BA-AW; Table S1: BBSS mass difference with different deashing treatments; Figure S2: SEM-EDS images of BA-WA at different temperatures; Figure S3: SEM-EDS images of BA-AW at different temperatures.
