# Supplementary material for: Effect of Deashing Treatment on Ash Fusion Characteristics of Biochar from Bamboo Shoot Shells
Source: Molecules. 2024 Mar 21;29(6):1400. doi: 10.3390/molecules29061400 (PMC10974987; doi:10.3390/molecules29061400)
Supplement: Supplementary file 1 [file molecules-29-01400-s001.zip › molecules-2882596-supplementary.pdf]

# Supplement materials

# Effect of Deashing Treatment on Ash Fusion Characteristics of Biochar from Bamboo Shoot Shells

**Hao Ren [1,2], Qi Gao [1,2], Liangmeng Ni [1,2], Mengfu Su [1,2], Shaowen Rong [1,2], Shushu Liu [1,2], Yanhang Zhong [1,2] and Zhijia Liu [1,2,*]**

[1] International Centre for Bamboo and Rattan, Beijing 100102, China; hao991105@163.com (H.R.); gaoqi677@163.com (Q.G.); 17346610822@163.com (L.N.); smf13850202763@163.com (M.S.); bramble_001@163.com (S.R.); ls2535075602@163.com (S.L.); zyh1842675310@gmail.com (Y.Z.)

[2] Key Laboratory of National Forestry and Grassland Administration/Beijing for Bamboo & Rattan Science and Technology, Beijing 100102, China

* Correspondence: liuzj@icbr.ac.cn

**Table S1** BBSS mass difference with different deashing treatments

| | Mass before deashing (g) | Mass after deashing (g) | Mass difference (g) |
|---|---|---|---|
| BBSS−WA | 10 | 8.857 | 1.143 |
| BBSS−AW | 10 | 8.983 | 1.017 |

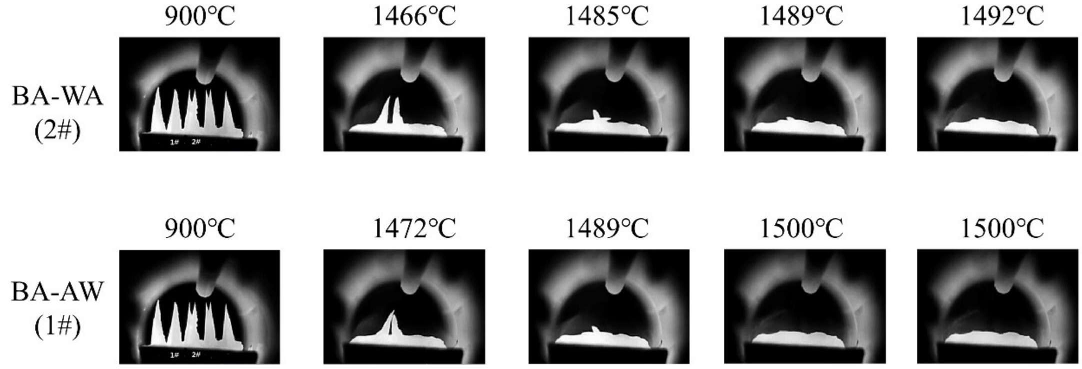


**Figure S1.** Ash fusion temperature of BA-WA and BA-AW.

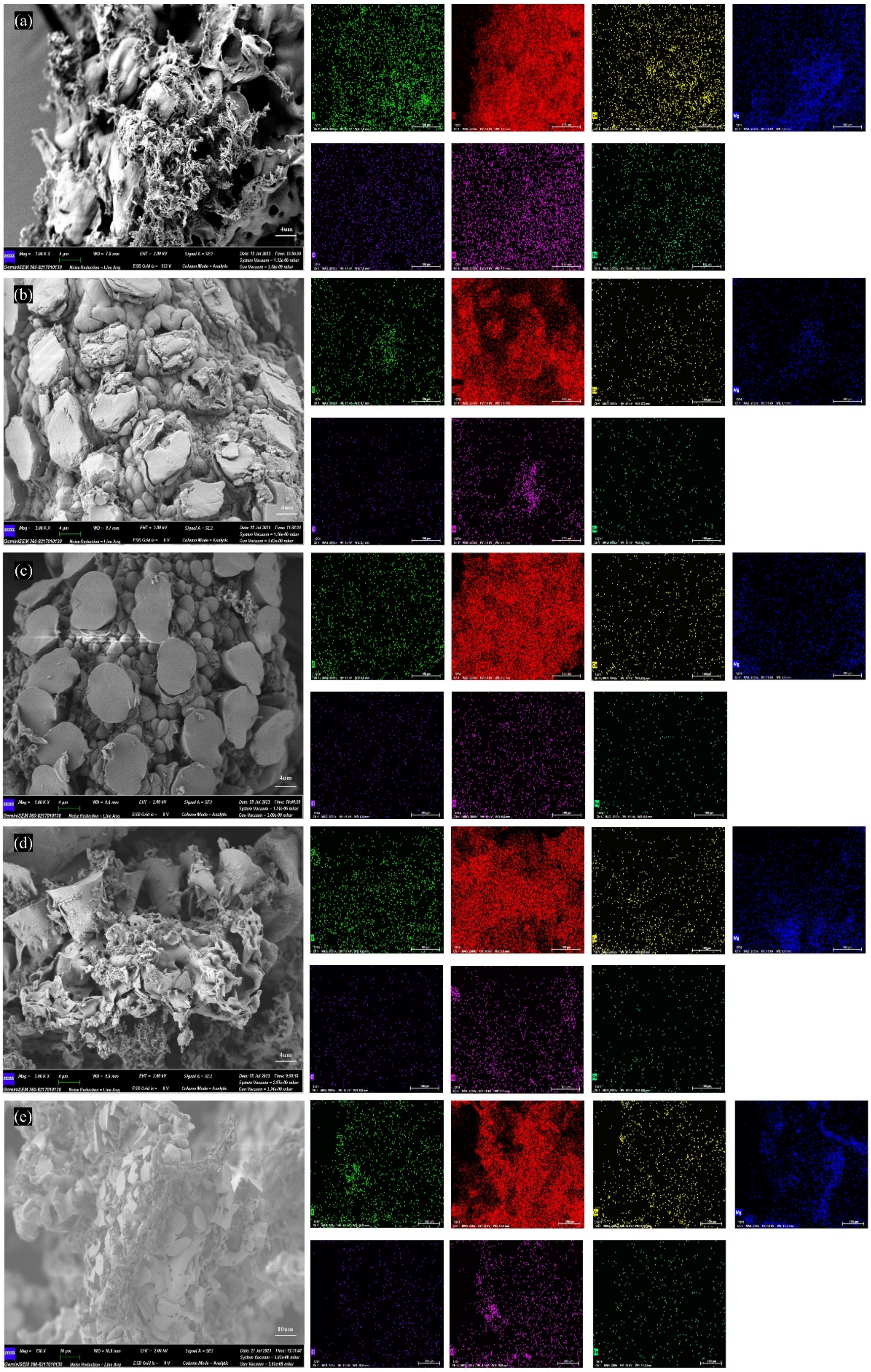


**Figure S2.** SEM-EDS images of BA-WA at different temperatures.

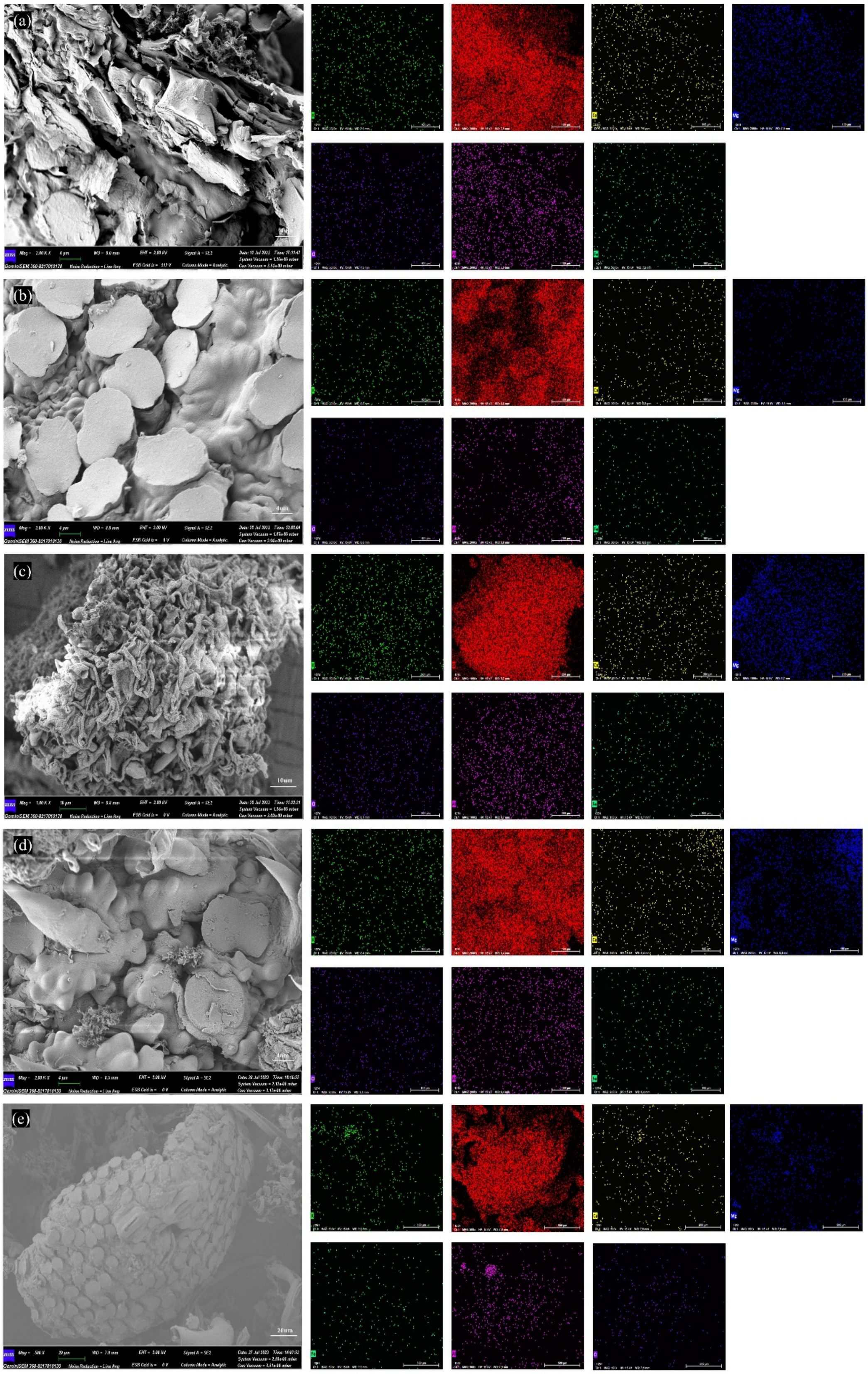

**Figure S3.** SEM-EDS images of BA-AW at different temperatures.
